# Association of the Triglyceride–Glucose Index with Ambulatory Blood Pressure Categories and Circadian Blood Pressure Phenotypes in Untreated Adults: A Single-Centre, ABPM-Based Retrospective Cross-Sectional Study

**DOI:** 10.3390/diagnostics16172754

**Published:** 2026-08-27

**Authors:** Mert Deniz Savcilioglu, Nil Savcilioglu, Osman Buyukcelebi, Irfan Veysel Duzen, Ertan Vuruşkan, Mehmet Murat Sucu

**Affiliations:** Cardiology Department, Faculty of Medicine, Gaziantep University Şahinbey Education and Research Hospital, 27310 Gaziantep, Türkiye; ncelikkalkan@gmail.com (N.S.); dr.buyukcelebi02@gmail.com (O.B.); vduzen79@yahoo.com.tr (I.V.D.); ertanvuruskan@hotmail.com (E.V.); mmuratsucu@gmail.com (M.M.S.)

**Keywords:** ambulatory blood pressure monitoring, triglyceride–glucose index, hypertension, circadian blood pressure phenotype, ordinal logistic regression

## Abstract

**Background/Objectives:** The triglyceride–glucose (TyG) index, a surrogate marker associated with insulin resistance, has been linked to both blood pressure elevation and circadian blood pressure dysregulation; however, its relationship with ambulatory blood pressure (BP) phenotypes across the full spectrum of contemporary ambulatory BP categories remains incompletely characterised. This study investigated the association of the TyG index with ambulatory BP categories and circadian BP phenotypes in untreated adults undergoing ambulatory blood pressure monitoring (ABPM). **Methods:** This retrospective cross-sectional study included 723 consecutive adults with no current or previous exposure to antihypertensive medication (treatment-naïve status refers exclusively to antihypertensive pharmacotherapy) who underwent 24 h ABPM and same-day laboratory assessment at a tertiary referral centre. Participants were classified according to the 2024 European Society of Cardiology ambulatory BP thresholds into non-elevated BP (*n* = 199), elevated BP (*n* = 243), or hypertension (*n* = 281) groups. Circadian BP phenotypes were categorised as extreme-dippers, dippers, non-dippers, and reverse-dippers. Associations between the TyG index and ambulatory BP categories were evaluated using multivariable proportional-odds ordinal logistic regression. The joint effect of BP category and circadian phenotype was examined by adding a TyG index × circadian phenotype interaction term to the ordinal model, and circadian phenotypes were characterised using multinomial logistic regression. **Results:** The TyG index increased progressively across ambulatory BP categories (8.8 ± 0.5, 9.1 ± 0.5, and 9.2 ± 0.6; *p*-trend < 0.001). After adjustment for age, sex, HbA1c, estimated glomerular filtration rate, and body mass index, each one-standard-deviation increase in the TyG index was associated with higher ambulatory BP category (OR 1.57, 95% CI 1.34–1.83; *p* < 0.001). The Brant test provided no evidence against the proportional-odds assumption (*p* = 0.379). In an exploratory analysis, the TyG–BP category association differed across circadian phenotypes (TyG × phenotype interaction, likelihood-ratio χ^2^ = 8.26, df = 3, *p* = 0.041). In multinomial analysis, a higher TyG index was associated with the reverse-dipper (OR 2.90 per SD, 95% CI 2.06–4.08) and extreme-dipper (OR 2.77, 95% CI 1.87–4.10) phenotypes relative to the results for the dippers (both *p* < 0.001), but not with non-dipping (OR 1.02, 95% CI 0.84–1.24; *p* = 0.86) after multivariable adjustment. **Conclusions:** Among untreated adults undergoing ABPM, higher TyG index values were associated with progressively higher ambulatory BP categories and with reverse- or extreme-dipper circadian BP phenotypes after multivariable adjustment. Because insulin resistance was not measured directly, these results describe an association between the TyG index, a surrogate marker, and ambulatory BP phenotypes; they should be interpreted as hypothesis-generating because of the retrospective cross-sectional design. Prospective studies are warranted to determine their temporal and clinical significance.

## 1. Introduction

Hypertension affects approximately 1.28 billion adults worldwide and remains among the leading modifiable contributors to cardiovascular morbidity and mortality [1]. A substantial proportion of individuals at elevated cardiovascular risk remain undetected under conventional office-based measurement strategies, underscoring the diagnostic value of ambulatory blood pressure monitoring (ABPM) [2,3]. ABPM provides a continuous haemodynamic profile over a full circadian cycle, captures phenomena such as white-coat and masked hypertension, and yields prognostic information that is superior to office measurement alone [3,4]. The 2024 European Society of Cardiology (ESC) guidelines formalised a three-tier ambulatory classification based on 24 h mean blood pressure—non-elevated (<115/65 mmHg), elevated (115–129/65–79 mmHg), and hypertension (≥130/80 mmHg)—thereby extending the phenotypic range of interest beyond overt hypertension to include the earlier elevated stratum [2]. This refined framework creates an opportunity to examine whether metabolic correlates differ across the ambulatory blood pressure continuum, including in individuals who would not be classified as hypertensive by conventional thresholds.

Beyond mean blood pressure level, the circadian pattern of blood pressure regulation carries additional prognostic significance. Current classifications recognise four circadian phenotypes based on the percentage decline in nocturnal systolic blood pressure relative to daytime values: dipper (10–20%), non-dipper (0–<10%), reverse-dipper (<0%), and extreme-dipper (>20%) [5]. Non-dipping, defined as an attenuated nocturnal systolic decline of less than 10%, has been associated with increased cardiovascular risk, target-organ damage, and all-cause mortality, over and above the risk associated with mean ambulatory blood pressure values [5,6]. Reverse-dipping, in which nocturnal pressure exceeds daytime values, has been linked to the highest cardiovascular event rates among all circadian categories in both general and chronic-kidney-disease populations, and is increasingly recognised as a distinct high-risk phenotype rather than as a variant of non-dipping [6,7]. Extreme-dipping, conversely, has been associated with silent cerebrovascular disease and nocturnal ischaemic events, although the data are less consistent [5]. Consequently, analyses restricted to the binary dipper/non-dipper classification may overlook clinically meaningful heterogeneity at the extremes of the nocturnal blood pressure spectrum.

The mechanisms underlying circadian blood pressure dysregulation are heterogeneous and incompletely understood; proposed contributors include dysregulation of the autonomic nervous system, overactivation of the renin–angiotensin–aldosterone system, impaired renal sodium handling, and heightened nocturnal sympathetic tone [8]. Obstructive sleep apnoea, through intermittent hypoxia and sympathetic activation, has also been identified as an important determinant of the non-dipping and reverse-dipping phenotypes, although its contribution may be difficult to disentangle from other inflammatory and metabolic processes in observational settings [9]. Systemic inflammation, reflected by markers such as C-reactive protein (CRP), has been reported to be higher in untreated hypertensive patients with a non-dipping pattern than in dippers in ABPM-based studies [10], although whether this association extends to the reverse- and extreme-dipper phenotypes has been less frequently examined.

Insulin resistance is a recognised pathophysiological correlate of hypertension. Through impairment of nitric oxide-mediated vasodilation, enhancement of renal sodium reabsorption, and upregulation of sympathoadrenal activity, insulin resistance has been proposed to contribute to elevated vascular tone and blood pressure [11]. The triglyceride–glucose (TyG) index, calculated as the natural logarithm of the product of fasting triglycerides and fasting glucose divided by two, has been proposed as a surrogate marker of insulin resistance that does not require insulin measurement [12,13]. Cross-sectional and longitudinal studies have reported associations between the TyG index and blood pressure elevation, and a meta-analysis of prospective cohorts found that higher TyG index values were associated with incident hypertension [14,15]. However, most existing work has relied on office blood pressure measurements; whether the TyG index is associated with blood pressure elevation across ambulatory blood pressure categories, including the full ordered spectrum, from non-elevated through elevated to hypertension as defined by the 2024 ESC classification, has not been comprehensively evaluated [2]. Moreover, most studies have examined metabolic markers in relation to binary hypertension status or to the dipper/non-dipper distinction separately; few have simultaneously assessed whether such markers show different patterns of association across both the ambulatory blood pressure continuum and the full range of circadian phenotypes within a single ABPM-based framework.

The present cross-sectional study examined whether the TyG index was associated with ambulatory blood pressure category, modelled as an ordered progression from non-elevated through elevated blood pressure to hypertension, and with circadian blood pressure phenotype in a cohort of 723 consecutive treatment-naïve adults undergoing 24 h ABPM. All four circadian phenotypes were retained in the analytical cohort, and ambulatory blood pressure classification followed the 2024 ESC 24 h thresholds [2]. Associations were evaluated using multivariable models adjusted for demographic, glycaemic, renal, and anthropometric covariates.

## 2. Materials and Methods

### 2.1. Study Design and Setting

This was a single-centre, retrospective, cross-sectional observational study conducted at the Cardiology Outpatient Clinic of Gaziantep University Şahinbey Education and Research Hospital, Gaziantep, Turkey. Ethical approval was obtained prior to data access and analysis (Gaziantep University Clinical Research Ethics Committee; Protocol No. 2026/251; approval date: 8 April 2026). Following ethical approval, electronic medical records were reviewed retrospectively to identify consecutive patients who had undergone 24 h ambulatory blood pressure monitoring (ABPM) between 1 January 2024 and 31 December 2025 and who met the predefined eligibility criteria. All data were fully anonymised before analysis. The study was conducted in accordance with the Declaration of Helsinki. Given the retrospective design and the use of anonymised data, the requirement for written informed consent was waived by the institutional ethics committee.

### 2.2. Study Population

Inclusion criteria. Patients were eligible if they were aged between 18 and 85 years and had been referred to the cardiology outpatient clinic for ABPM evaluation. Eligibility additionally required a complete ABPM recording with interpretable daytime and nighttime mean values and the availability of same-day haematological and biochemical laboratory measurements obtained concurrently with ABPM placement. All included individuals were treatment-naïve, with no prior diagnosis of hypertension and no exposure to antihypertensive medications at the time of ABPM. Treatment-naïve status refers specifically to antihypertensive pharmacotherapy. Use of other cardiovascular or metabolic medications (for example statins, metformin, SGLT2 inhibitors or GLP-1 receptor agonists) was not systematically recorded in the archived outpatient records and could not be reliably ascertained; such medication use could therefore neither be applied as an exclusion criterion nor entered as a covariate. This reflects the limits of the archived record rather than a decision to retain known users, and is acknowledged in the Limitations section.

**Figure 1:**
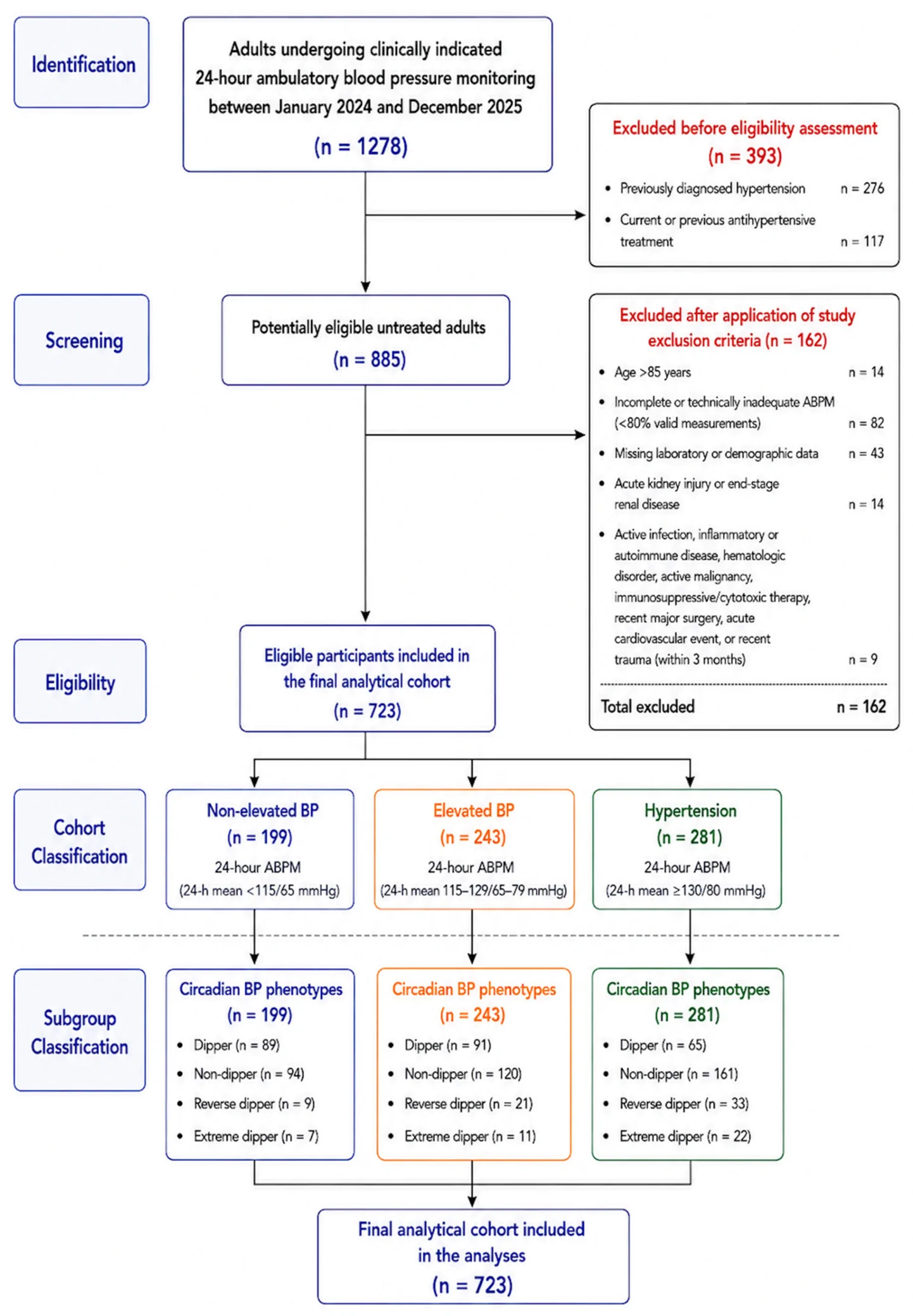
STROBE flow diagram illustrating participant selection, cohort derivation, ambulatory blood pressure classification, and circadian blood pressure phenotyping. The study is reported in accordance with the Strengthening the Reporting of Observational Studies in Epidemiology (STROBE) statement [16]. During the study period, 1278 adults underwent clinically indicated 24 h ambulatory blood pressure monitoring; after exclusion of 393 individuals with prior hypertension or antihypertensive treatment and a further 162 who did not meet the eligibility criteria, 723 participants were included in the final analytical cohort. Participants were categorised as Non-Elevated BP (*n* = 199), Elevated BP (*n* = 243), or Hypertension (*n* = 281), according to the 2024 ESC ambulatory BP classification, and were subsequently stratified into dipper, non-dipper, reverse-dipper, and extreme-dipper circadian BP phenotypes [2].

Exclusion criteria. Patients were excluded if they had a prior or current diagnosis of hypertension or were receiving, or had previously received, any antihypertensive therapy. Individuals with conditions known to confound inflammatory biomarkers were excluded, including: active infection or acute inflammatory illness; active malignancy or ongoing oncological treatment; pregnancy; established chronic inflammatory or autoimmune diseases (rheumatoid arthritis, systemic lupus erythematosus, inflammatory bowel disease); haematological disorders; and current use of corticosteroids, immunosuppressants, or cytotoxic agents. Further exclusion criteria included acute kidney injury or end-stage renal disease; major surgery, acute cardiovascular event, or trauma within the preceding three months; ABPM recordings with fewer than 80% valid measurements; and missing laboratory or demographic data. The initial count of 1278 refers to unique individuals rather than to ABPM studies: every patient identified in the source registry underwent a single 24 h ABPM examination during the study period, and no individual contributed more than one recording; consequently, no repeated recordings had to be discarded. Each participant, therefore, contributes exactly one ABPM recording and one observation to the analysis. Exclusion criteria were applied sequentially in the order shown in Figure 1, and each excluded patient is counted once only, under the first criterion met; the categories in Figure 1 are therefore mutually exclusive.

A total of 1278 adults underwent clinically indicated ABPM during the study period. Of these, 393 were excluded before eligibility assessment because of a prior or current diagnosis of hypertension (*n* = 276) or current or previous antihypertensive treatment (*n* = 117), leaving 885 potentially eligible treatment-naïve adults. A further 162 were excluded by the eligibility criteria detailed above (itemised in Figure 1), yielding a final analytical cohort of 723 participants. The final exclusion category in Figure 1 (*n* = 9) aggregates the remaining clinical criteria—active infection or acute inflammatory illness; active malignancy or ongoing oncological treatment; established chronic inflammatory or autoimmune disease; and major surgery, acute cardiovascular event or trauma within the preceding three months. These are reported as a single composite category because the individual counts are small enough that separate reporting could permit re-identification in a single-centre cohort; the criteria were nonetheless applied sequentially, and each patient was counted once under the first criterion met. The 43 participants excluded for missing data are itemised by variable in Supplementary Table S9. The flow of participant selection, exclusions, and phenotype assignment is summarised in the STROBE flow diagram (Figure 1).

### 2.3. Ambulatory Blood Pressure Monitoring

Twenty-four-hour ambulatory blood pressure monitoring (ABPM) was performed using an oscillometric ambulatory blood pressure monitor (Echo80^®^, Borsam Biomedical Instruments, Shenzhen, China), operated according to standard ABPM methodology. The AAMI/ESH/ISO Universal Standard [17] defines the general protocol for validating blood-pressure-measuring devices, but this does not by itself constitute an independent, device-specific validation of this monitor; the absence of such a validation study for the Echo80 is acknowledged as a limitation. All ambulatory blood pressure recordings were acquired and processed using the manufacturer’s proprietary software under routine clinical practice conditions. Appropriate cuff sizes were selected according to individual arm circumference, and cuff position and signal quality were verified at the time of device placement, in accordance with the manufacturer’s standard operating procedure. A standardised office blood pressure measurement was not part of the routine ABPM protocol at this centre; office blood pressure measurement was therefore not performed for study purposes, was not available in the archived records, and could not be reported as a study variable. Heart-rate summaries were likewise not consistently retained in the archived ambulatory reports and could not be tabulated. Blood pressure measurements were scheduled every 15 min during the daytime (06:00–22:00) and every 30 min during the nighttime (22:00–06:00). Participants were instructed to maintain their usual daily activities and to keep the monitored arm still during measurements. Recordings with at least 80% valid measurements across the whole 24 h period were considered technically acceptable for analysis; readings flagged as artefacts by the device software were not counted as valid. Separate minimum requirements for the number of valid daytime and nighttime readings were not applied by the local protocol, and neither the total nor the period-specific number of valid readings per participant could be recovered from the archived reports. Daytime and nighttime windows were defined by fixed clock times because sleep–wake diaries and actigraphy were not part of the routine protocol, and information on shift work or atypical sleep schedules was not recorded. These constraints are set out in the Limitations section.

The device software automatically generated all ambulatory blood pressure parameters, including 24 h, daytime, nighttime, morning surge, morning peak, and maximum/minimum blood pressure values, from all valid recordings, without manual recalculation. Of these, only the 24 h, daytime and nighttime mean systolic and diastolic values were extracted and analysed; morning surge, morning peak and maximum/minimum values were produced by the device software but were not retained in a uniform format across the archived reports, were not analysed, and are therefore neither reported nor interpreted here. Participant classification was based exclusively on the reported 24 h mean systolic and diastolic blood pressure values. The 24 h mean was computed by the device software as a time-weighted average of all valid daytime and nighttime readings, rather than as a simple arithmetic mean of all measurements.

Blood pressure classification followed the 2024 European Society of Cardiology (ESC) guidelines using 24 h mean ambulatory blood pressure thresholds [2]: non-elevated BP was defined as 24 h mean systolic BP <115 mmHg and diastolic BP <65 mmHg; elevated BP as systolic BP 115–129 mmHg and/or diastolic BP 65–79 mmHg; and hypertension as systolic BP ≥130 mmHg and/or diastolic BP ≥80 mmHg. Three mutually exclusive groups were formed: non-elevated BP, elevated BP, and hypertension.

When systolic and diastolic blood pressure values corresponded to different ambulatory blood pressure categories, classification was based on the higher category, consistent with the 2024 ESC recommendations [2].

Circadian blood pressure phenotypes were classified according to the percentage decline in mean nighttime systolic blood pressure relative to mean daytime systolic blood pressure: extreme-dipper (>20%), dipper (10–20%), non-dipper (0–<10%), and reverse-dipper (<0%). All four phenotypes were retained in the analytical cohort. As all participants were untreated at the time of assessment, the observed ambulatory blood pressure profiles and circadian patterns were not influenced by antihypertensive pharmacotherapy.

### 2.4. Laboratory Measurements

Venous blood samples were obtained after overnight fasting, concurrently with ABPM placement. Haematological parameters, including white blood cell differential counts, were measured using an automated analyser (Sysmex XN-1000, Sysmex Corporation, Kobe, Japan). Biochemical parameters—including fasting glucose, triglycerides, HDL-cholesterol (HDL-C), creatinine, glycated haemoglobin (HbA1c), and C-reactive protein (CRP)—were analysed using the Siemens Atellica^®^ analytical platform (Siemens Healthineers, Erlangen, Germany) in the hospital’s central clinical biochemistry laboratory, according to the manufacturer’s standard operating procedures and institutional quality-control protocols. Serum CRP was measured on the same platform as part of routine clinical laboratory testing; a high-sensitivity CRP assay was not uniformly used, and CRP was therefore treated as a descriptive variable only and not entered into the multivariable models; accordingly, CRP values should not be interpreted as though derived from a single homogeneous assay. The estimated glomerular filtration rate (eGFR) was calculated using the Chronic Kidney Disease Epidemiology Collaboration (CKD-EPI) 2021 creatinine equation [18].

### 2.5. Derived İndices

The triglyceride–glucose (TyG) index, an indirect surrogate marker of insulin resistance, was calculated as: TyG = ln [triglycerides (mg/dL) × fasting glucose (mg/dL)/2] [12]. Body mass index (BMI) was calculated as weight in kilograms divided by height in metres squared (kg/m^2^). Diabetes mellitus was defined as a fasting glucose ≥126 mg/dL and/or HbA1c ≥6.5% and/or a pre-existing physician’s diagnosis documented in the medical record.

### 2.6. Statistical Analysis

All analyses were performed in R (version 4.3.3; R Foundation for Statistical Computing, Vienna, Austria). A two-sided *p* < 0.05 was considered statistically significant. The complete analysis script, data dictionary, file manifest, session log (R version, package versions and random seeds), an environment lock file, the exact machine-generated aggregate outputs of the source-data run, and a synthetic dataset that permits the full workflow to be executed end to end are openly available at https://doi.org/10.5281/zenodo.21951990.

#### 2.6.1. Descriptive Statistics

Continuous variables are reported as mean ± SD when approximately normally distributed and as the median [interquartile range (IQR)] when skewed. The choice of summary measure was guided by the Shapiro–Wilk test and visual inspection of the distributions. Overall comparisons across the three blood pressure categories used one-way ANOVA (normally distributed), the Kruskal–Wallis test (skewed), or the Chi-square test (categorical). Tests for monotonic trends across the ordered categories (non-elevated → elevated → hypertension) used the Jonckheere–Terpstra test for continuous variables and the Cochran–Armitage test for proportions. Post hoc pairwise comparisons used Tukey’s HSD for normally distributed variables and the Dunn test with Bonferroni correction for skewed variables (Table S2).

**Figure 2:**
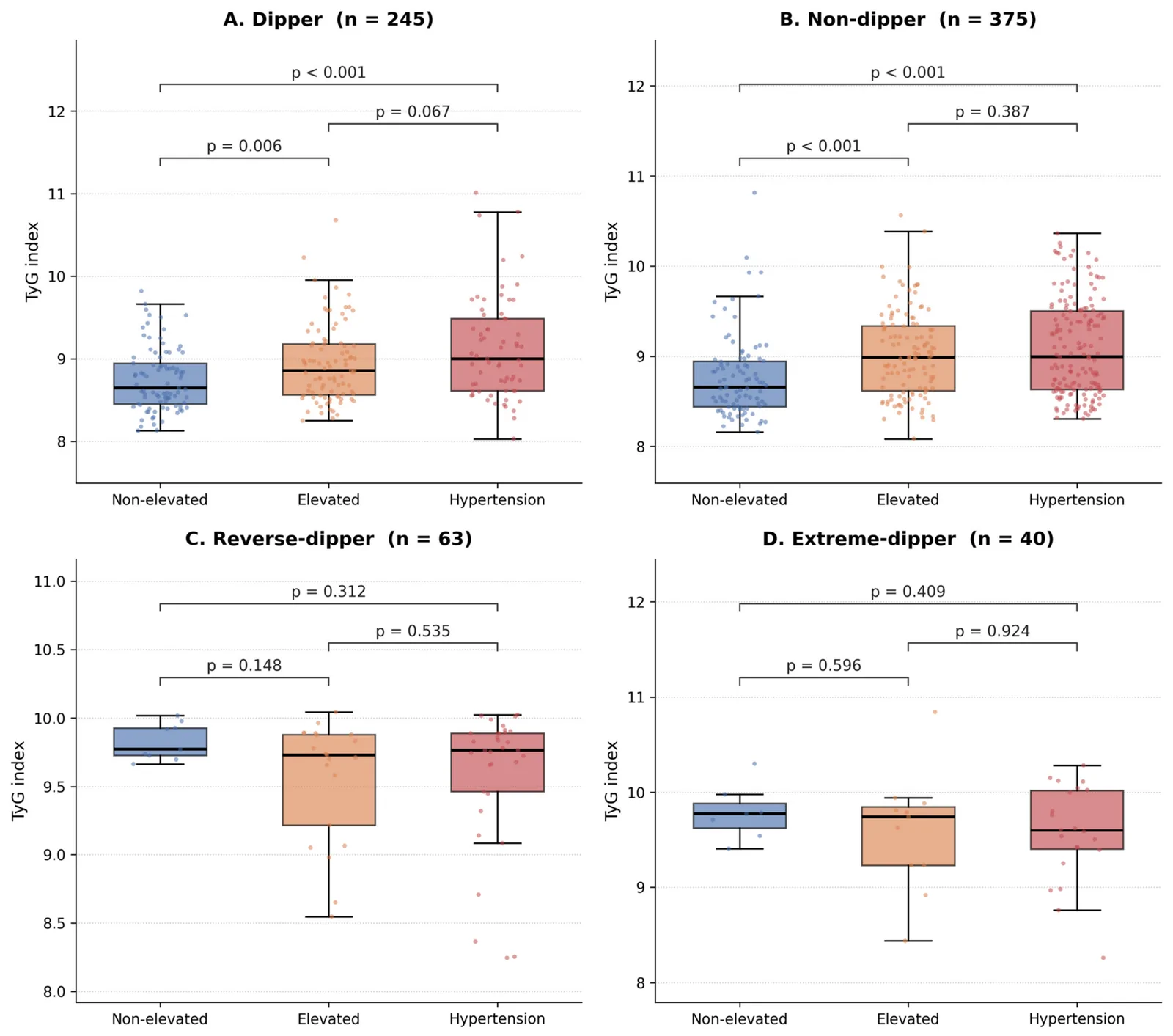
TyG index across ambulatory BP categories within each circadian phenotype. Distribution of the TyG index across the three ambulatory BP categories (Non-Elevated, Elevated, Hypertension) within each circadian phenotype: (**A**) dipper; (**B**) non-dipper; (**C**) reverse-dipper; (**D**) extreme-dipper. Boxes show median and IQR; whiskers, the 1.5 × IQR range; and points, individual patients. Brackets show all three exploratory pairwise comparisons between ambulatory BP categories within each phenotype (Mann–Whitney U test, uncorrected), with the corresponding *p*-value shown above each bracket.

The metabolic profile across the four circadian phenotypes within each blood pressure category was compared using one-way ANOVA or the Kruskal–Wallis test, as appropriate. Post hoc pairwise contrasts are presented as a compact letter display (Tukey’s HSD for TyG index and HDL-C; Dunn–Bonferroni for skewed variables). Pairwise comparisons of the TyG index across blood pressure categories within each phenotype used the Mann–Whitney U test (Figure 2). These 12 comparisons are descriptive, were not corrected for multiplicity, and several of them involve very small phenotype-by-category cells; a non-significant result in those panels reflects limited precision and is not evidence that the categories do not differ. To allow that distinction to be made quantitatively, the same 12 between-category comparisons are also reported as model-based adjusted mean differences with 95% confidence intervals, obtained from a linear model of the TyG index containing an ambulatory BP category × circadian phenotype interaction and adjusted for age, sex, eGFR and BMI (Supplementary Table S11).

Bivariate associations between the TyG index and metabolic and renal variables were assessed by Spearman rank correlation (Table S1).

#### 2.6.2. Ordinal Logistic Regression

Because the three blood pressure categories form a natural ordering (non-elevated < elevated < hypertension), a proportional-odds ordinal logistic regression model was fitted as the primary analysis, using all 723 participants simultaneously. The model included age, sex, HbA1c, eGFR, BMI, and the TyG index; continuous predictors were expressed per one-standard-deviation (SD) increment to facilitate comparison across variables. The target quantity was defined explicitly as a descriptive multivariable (conditional) association between the TyG index and the ordered ambulatory BP category; it is neither a total causal effect nor a controlled direct effect. This choice is deliberate. Age and sex can reasonably be regarded as common causes of both the TyG index and blood pressure, whereas HbA1c, diabetes, adiposity, and renal function are not unambiguously antecedent to the exposure and may lie on, or share a common source with, the pathway between insulin resistance and blood pressure. Correlation with both exposure and outcome does not by itself establish confounder status, and conditioning on a variable downstream of the exposure can induce over-adjustment. The assumed structure, the variables conditioned on, and the principal unmeasured variables are therefore displayed in a conceptual causal diagram (Supplementary Figure S1) [19]; because that structure cannot be verified in cross-sectional data, the resulting odds ratios are reported as conditional associations only and are given no causal interpretation. To make the adjustment strategy transparent rather than to assert that any particular variable is a confounder, the primary model was built up as a nested sequence: the TyG index alone; with age and sex; with renal function and adiposity added; and finally, with HbA1c added as a potentially intermediate metabolic variable (Supplementary Table S10). A further model replacing HbA1c with the binary diabetes indicator is reported in the same table. The proportional-odds (parallel-lines) assumption was evaluated using the Brant test [20] (Supplementary Table S7).

#### 2.6.3. Interaction Analysis (Exploratory)

Whether or not the association between the TyG index and BP category varied across circadian phenotypes was examined by adding a TyG index × circadian phenotype interaction term to the ordinal model described above; the interaction was assessed via a likelihood-ratio test. The TyG index was standardised (mean 0, SD 1) before the product terms were formed so that the phenotype main effects in the expanded model are interpretable at the cohort mean TyG value. This analysis was not defined before the data were examined, and no claim of prior specification is made; it is reported as exploratory. The proportional-odds assumption was evaluated separately for this expanded model, using the same Brant procedure applied to the primary model (Supplementary Table S7, panel B).

#### 2.6.4. Circadian Phenotype Analysis (Secondary)

Associations between the TyG index and circadian phenotype were examined using multinomial logistic regression, with the four circadian phenotypes as the outcome (reference: dipper), adjusted for age, sex, BMI, eGFR, HbA1c, and blood pressure category (Table S5). Departure from linearity was assessed in the direction of the scientific question, that is, with the circadian outcome as the dependent variable and the TyG index as the exposure. First, the continuous nocturnal systolic dipping percentage was modelled as the outcome, with the TyG index entered linearly, then as a quadratic term, and then as a restricted cubic spline, with three knots placed at the 10th, 50th and 90th percentiles of the TyG distribution; non-linearity was assessed by the significance of the additional term and by the corresponding F test (Supplementary Table S8, panels A and B). Second, because the scientific aim is the categorical phenotype, a multinomial model containing the same flexible TyG term was compared with the corresponding linear model via a likelihood-ratio test (Supplementary Table S8, panel C). A reverse-direction model, in which the TyG index is the outcome and continuous dipping the exposure, is additionally reported (Supplementary Table S8, panel D). Conditional regressions are not symmetric, so that panel describes only how the mean TyG index varies across the dipping spectrum; it does not test the non-linearity of the primary estimand, it is presented as descriptive, and it is not used to justify the categorical treatment of circadian phenotype. All models were adjusted for age, sex, HbA1c, eGFR, BMI, and ambulatory BP category. These analyses are secondary.

#### 2.6.5. Sensitivity Analysis

To examine whether the observed associations were driven by participants with established diabetes, the primary ordinal analysis was repeated after excluding patients with diabetes mellitus (*n* = 496 remaining; Table S3). No quantitative bias analysis of unmeasured confounding was performed. The assumptions required to place an ordinal common odds ratio on a risk-ratio scale could not be justified for this estimand, so the potential influence of unmeasured factors—in particular, obstructive sleep apnoea and metabolic therapies, neither of which was recorded—is addressed qualitatively, rather than quantified, in the Limitations section.

## 3. Results

### 3.1. Study Population and Cohort Derivation

During the study period, 1278 adults underwent clinically indicated 24 h ABPM; after exclusion of 393 individuals with prior hypertension or antihypertensive treatment (leaving 885 treatment-naïve adults) and a further 162 who did not meet the eligibility criteria, 723 participants formed the final analytical cohort; the derivation is detailed in the STROBE flow diagram (Figure 1). Participants were classified according to the 2024 ESC ambulatory BP thresholds into Non-Elevated BP (*n* = 199), Elevated BP (*n* = 243), and Hypertension (*n* = 281) groups. Missing data before complete-case analysis were infrequent: of the 885 potentially eligible participants, 43 (4.9%) had a missing study variable, the individual variable-specific counts sum to exactly 43 so that no participant had more than one missing value, and no single variable was missing in more than 2% of records (Supplementary Table S9). A formal comparison of included with excluded participants was not undertaken because the records of excluded individuals were incomplete for the variables that such a comparison would require.

Baseline clinical, laboratory, and haemodynamic characteristics across blood pressure categories are presented in Table 1. Age increased across categories (46.6 ± 15.3, 49.8 ± 16.0, and 55.3 ± 13.9 years; *p* < 0.001, *p*-trend < 0.001), as did the proportion of men (34.2%, 39.5%, 44.1%; *p*-trend = 0.028) and the prevalence of diabetes (24.1%, 30.5%, 37.4%; *p* = 0.008). Body-mass index was comparable across categories (*p* = 0.325). Fasting glucose (91.0 [82.0–103.0] to 107.0 [90.0–131.0] mg/dL) and triglycerides both increased across categories (both *p* < 0.001), whereas HDL-C decreased (47.4 ± 6.9 to 45.7 ± 7.2 mg/dL; *p* = 0.030). eGFR showed a downward gradient (103.0 ± 20.4 to 95.7 ± 18.7 mL/min/1.73 m^2^; *p* < 0.001), while CRP differed only by trend (2.0 [0.8–4.6] to 2.8 [1.2–5.4] mg/L; *p* = 0.054, *p*-trend = 0.016). The TyG index increased across categories (8.8 ± 0.5, 9.1 ± 0.5, and 9.2 ± 0.6; *p* < 0.001, *p*-trend < 0.001), with all three pairwise contrasts significant (Table S2). Twenty-four-hour, daytime, and nighttime systolic and diastolic BP values increased progressively across the three ambulatory BP categories (all *p* < 0.001). The nocturnal systolic dipping percentage decreased across categories (median 9.8%, 8.3%, and 5.1%; *p* < 0.001, *p*-trend < 0.001).

### 3.2. Circadian Phenotype Distribution and Metabolic Profile

The four circadian phenotypes were distributed unevenly across BP categories (χ^2^, *p* < 0.001; Table S4). Considered separately, reverse dippers represented 4.5%, 8.6% and 11.7%, and extreme-dippers 3.5%, 4.5% and 7.8%, of the non-elevated, elevated and hypertension categories, respectively (Table S4). These proportions are descriptive; the two phenotypes are modelled separately throughout and are not combined into a single category at any point in the analysis.

Within each BP category, the metabolic profile of the four phenotypes is shown in Table 2. In every stratum, the TyG index was higher in the reverse- and extreme-dipper subgroups than for dippers and non-dippers (non-elevated BP 9.8 vs. 8.8; elevated BP 9.6 vs. 8.9–9.0; hypertension 9.6 vs. 9.1; overall *p* < 0.001 in each stratum). Similar differences were observed for fasting glucose and triglycerides (both *p* < 0.001 within each stratum, except triglycerides in the hypertension group, *p* = 0.019). HDL-C was lowest in the extreme-dipper subgroup within each category (*p* ≤ 0.007). CRP differed across phenotypes only within the hypertensive stratum (*p* = 0.014), where non-dippers carried the highest values; it did not differ across phenotypes in the non-elevated or elevated strata (*p* = 0.123 and *p* = 0.236).

The distribution of the TyG index across BP categories within each circadian phenotype is displayed in Figure 2. In the dipper and non-dipper panels, the index increased across BP categories (non-elevated vs. hypertension, *p* < 0.001 in both; non-elevated vs. elevated, *p* = 0.006 and *p* < 0.001, respectively), whereas in the reverse- and extreme-dipper panels, the between-category differences were not statistically significant. All 12 comparisons are descriptive and uncorrected, and the corresponding cells contain as few as seven participants; the absence of significance in these panels is compatible with low precision and should not be read as evidence that the categories are homogeneous. The corresponding model-based adjusted mean differences make this explicit: within dippers and non-dippers, the adjusted TyG difference between the hypertension and non-elevated categories was +0.31 (95% CI +0.15 to +0.47) and +0.28 (+0.16 to +0.40), whereas within reverse- and extreme-dippers, the intervals were wide and included differences in both signs (−0.24, 95% CI −0.60 to +0.12; and −0.28, 95% CI −0.69 to +0.14; Supplementary Table S11).

### 3.3. TyG İndex and the Ordered BP-Category Progression

The TyG index correlated with triglycerides (ρ = +0.870) and fasting glucose (ρ = +0.686; both *p* < 0.001). Weaker correlations were observed with HbA1c (ρ = +0.265), HDL-C (ρ = −0.288), and eGFR (ρ = −0.253) (all *p* < 0.001), whereas no correlation was observed with BMI (ρ = +0.014, *p* = 0.699) (Table S1).

In a proportional-odds ordinal model of the ordered outcome (non-elevated < elevated < hypertension) adjusted for age, sex, HbA1c, eGFR, and BMI, each 1-SD increment in the TyG index remained associated with higher ambulatory BP category after multivariable adjustment (OR 1.57 per SD increase, 95% CI 1.34–1.83; *p* < 0.001). Age was also associated with higher ambulatory BP category (OR 1.49 per SD, 95% CI 1.23–1.79; *p* < 0.001), whereas HbA1c, eGFR, and BMI were not associated with the progression after adjustment (Table 3). The Brant test provided no evidence against the proportional-odds assumption (omnibus χ^2^ = 6.41, df = 6, *p* = 0.379).

The estimate was stable across the nested adjustment sequence: the per-SD odds ratio for the TyG index was 1.64 (95% CI 1.42–1.89) unadjusted, 1.51 (1.30–1.75) after adding age and sex, 1.53 (1.32–1.78) after further adding eGFR and BMI, and 1.57 (1.34–1.83) in the primary model that also includes HbA1c; replacing HbA1c with the binary diabetes indicator yielded 1.57 (1.34–1.86) (Supplementary Table S10). Conditioning on the metabolic variables whose status as confounder or intermediate is uncertain; therefore, these variables changed the estimate by less than 5%. Variance inflation factors were below 2 for all predictors (TyG index 1.16; Table S6), indicating no problematic multicollinearity among the model covariates.

### 3.4. Variation in the TyG–BP Association Across Circadian Phenotypes

Adding a TyG index × circadian phenotype interaction term to the ordinal model indicated that the association between the TyG index and BP category varied across circadian phenotypes (likelihood-ratio χ^2^ = 8.26, df = 3, *p* = 0.041). The incremental per-SD TyG effect was attenuated in reverse-dippers relative to dippers (interaction OR 0.45, 95% CI 0.24–0.88; *p* = 0.019), with a similar but non-significant pattern in extreme-dippers (OR 0.55, 95% CI 0.27–1.12; *p* = 0.101) and no difference in non-dippers (OR 0.96, 95% CI 0.68–1.35; *p* = 0.81; Table 4). Within the same model, the per-SD TyG association in the reference (dipper) phenotype was OR 1.69 (95% CI 1.28–2.21; *p* < 0.001), and each of the three remaining phenotypes was itself associated with a higher BP category (non-dipper OR 1.72, 95% CI 1.26–2.36; reverse-dipper OR 3.36, 95% CI 1.49–7.59; extreme-dipper OR 3.63, 95% CI 1.41–9.33; Table 4). The Brant test applied to this expanded model provided no evidence against the proportional-odds assumption (omnibus χ^2^ = 13.44, df = 12, *p* = 0.338; no individual term *p* < 0.18; Table S7, panel B). This interaction analysis is exploratory.

### 3.5. TyG İndex and Circadian Phenotype

In multinomial logistic regression with the four circadian phenotypes as the outcome (reference: dipper), adjusted for age, sex, BMI, eGFR, HbA1c, and BP category, a higher TyG index was associated with the reverse-dipper (OR 2.90 per SD, 95% CI 2.06–4.08; *p* < 0.001) and extreme-dipper (OR 2.77, 95% CI 1.87–4.10; *p* < 0.001) phenotypes, but not with non-dipping (OR 1.02, 95% CI 0.84–1.24; *p* = 0.86; Table S5). With the continuous nocturnal systolic dipping percentage as the outcome and the TyG index as the exposure, the linear association was weak and not statistically significant after adjustment (β = −0.67 per SD, 95% CI −1.45 to 0.12; *p* = 0.096). Departure from linearity in this direction was likewise not statistically significant: the restricted cubic spline non-linear term gave *p* = 0.063 (F = 3.46 on 1 and 713 df) and the quadratic term *p* = 0.623, with the model R^2^ rising only from 0.064 to 0.069 (Table S8, panel A). The corresponding adjusted mean dipping percentage varied only modestly across the observed TyG range (6.1% at a TyG index of 8.0, 7.8% at 9.0 and 3.3% at 10.5), and the confidence limits around the non-linear term include no departure from linearity, so no particular functional shape is claimed (Table S8, panel B). When the categorical phenotype was used as the outcome instead, adding the same flexible TyG term to the multinomial model did not improve fit over a linear term (likelihood-ratio χ^2^ = 0.79, df = 3, *p* = 0.853; Table S8, panel C). A reverse-direction model, with the TyG index as the outcome and continuous dipping as the exposure, is reported for description only in Table S8, panel D; because conditional regressions are not symmetric, it cannot be read as evidence about the non-linearity of the primary estimand.

### 3.6. Sensitivity Analysis

With the 227 participants with diabetes excluded (*n* = 496), the gradient in the TyG index across BP categories persisted (8.7, 8.9, and 9.0; *p* < 0.001), and each 1-SD increment remained associated with a higher BP category in the fully adjusted ordinal model (OR 1.45, 95% CI 1.22–1.73; *p* < 0.001; Table S3).

## 4. Discussion

In this cross-sectional study of 723 consecutive treatment-naïve individuals who underwent 24 h ABPM, the TyG index was associated with ambulatory blood pressure category across the ordered spectrum, from non-elevated, through elevated, to hypertension, after adjustment for age, sex, HbA1c, eGFR, and BMI. In a secondary multinomial analysis, a higher TyG index was associated with the reverse-dipper and extreme-dipper phenotypes, but not with non-dipping, relative to dippers, after adjustment for blood pressure category and metabolic covariates. The cross-sectional design precludes causal inference, and the findings should be interpreted as quantifying associations rather than as evidence for biological causality or clinical prediction.

The association between the TyG index and ambulatory blood pressure category is broadly consistent with prior evidence linking surrogate measures of insulin resistance to blood pressure elevation. A meta-analysis of prospective cohorts reported that higher TyG values were associated with incident hypertension [14], and a large multi-continent observational study found graded associations between TyG quartiles and both prevalent hypertension and cardiovascular mortality [15]. Most of this evidence, however, was derived from office blood pressure measurements, and data relating the TyG index specifically to ambulatory blood pressure parameters remain limited; higher TyG values have nonetheless been associated with adverse cardiovascular outcomes, including incident stroke, in hypertensive populations [21]. The present analysis extends these observations to a treatment-naïve cohort classified according to the 2024 ESC 24 h ambulatory thresholds [2], which include the intermediate elevated category. Using an ordinal model that retains all three categories in a single analysis, rather than discarding the elevated group or modelling two separate binary contrasts, avoids the information loss inherent in pairwise comparisons and allows evaluation of the gradient across the ambulatory spectrum [22].

The Brant test provided no evidence against the proportional-odds assumption, which is compatible with an association between the TyG index and blood pressure category that is approximately constant across both thresholds of the ordered outcome. This does not imply that the TyG index drives the transition from one category to the next in a biological sense; rather, it indicates that a single odds ratio adequately summarises the cross-sectional gradient. Among the adjusted covariates, age and the TyG index were associated with higher ambulatory blood pressure category, whereas HbA1c, eGFR, and BMI were not. The low multicollinearity across all predictors (VIF < 2) suggests that these variables provide complementary rather than redundant information in the adjusted model.

The exploratory interaction analysis indicated that the association between the TyG index and blood pressure category was not uniform across circadian phenotypes (likelihood-ratio *p* = 0.041). This pattern was visually apparent in the stratified boxplots: within the dipper and non-dipper subgroups, the TyG index increased across blood pressure categories, whereas within the reverse- and extreme-dipper subgroups—which carried uniformly elevated TyG values—no clear gradient across blood pressure categories was apparent. Those subgroups are small, and the underlying comparisons are descriptive, so the flatter appearance may reflect limited precision as much as genuine homogeneity. These observations do not permit conclusions about directionality or mechanism; they suggest that the relationship between insulin-resistance markers and ambulatory blood pressure is not uniform across circadian phenotypes and that analyses collapsing all phenotypes into a single stratum may obscure meaningful heterogeneity.

In the multinomial analysis, a higher TyG index was associated with the reverse- and extreme-dipper phenotypes but not with non-dipping; ambulatory blood pressure category itself remained associated with all three non-dipper phenotypes after adjustment for metabolic and renal covariates (Table S5). Because the TyG index is mathematically derived from fasting triglyceride and glucose concentrations, and participants with reverse- or extreme-dipper phenotypes already exhibited markedly higher values of both components, part of this association reflects the metabolic separation between circadian phenotypes rather than identifies a marker of nocturnal blood pressure behaviour specifically. The signal was therefore concentrated at both ends of the nocturnal dipping spectrum rather than along it, which is why a linear model of the continuous dipping percentage was correspondingly weak. Formal testing in the direction of the question did not, however, establish a non-linear relationship: neither a spline nor a quadratic term for the TyG index improved the model for continuous dipping, and a flexible TyG term did not improve the multinomial model for the categorical phenotype. The categorical treatment of circadian phenotype is retained because it is the classification used clinically and in the prognostic literature, not because a non-linear shape was demonstrated. These cross-sectional associations, based on modest numbers of participants in the reverse- and extreme-dipper subgroups (*n* = 63 and *n* = 40), should not be interpreted as evidence of clinical utility for identifying circadian phenotypes.

CRP was retained as a descriptive variable and was not entered into the multivariable models for two reasons, which can be stated transparently: a high-sensitivity assay was not used uniformly across the study period, so the CRP values are not assay-homogeneous, and the analysis was built around a single metabolic exposure, the TyG index, rather than around a combined inflammatory–metabolic model. In the descriptive analysis, CRP showed a modest trend across blood pressure categories (*p*-trend = 0.016) and differed across circadian phenotypes within the hypertensive stratum (*p* = 0.014). Whether or not inflammatory indices provide information beyond the TyG index for the characterisation of circadian blood pressure phenotypes remains an open question for adequately powered studies with longitudinal follow-up.

Several features of the study design support the internal validity of the findings. All participants were treatment-naïve with respect to antihypertensive pharmacotherapy, removing a major source of confounding for both ambulatory blood pressure profiles and circadian patterns. Blood pressure classification followed the 2024 ESC 24 h mean thresholds throughout, and all four circadian phenotypes were retained rather than excluded. The analytical approach used an ordinal model as the primary analysis and reported the proportional-odds assumption test, consistent with the natural ordering of the outcome [20]. The study is reported in accordance with the STROBE guidelines [16].

### Limitations

Several limitations warrant consideration. First, the cross-sectional design precludes causal inference. The direction of the observed associations cannot be determined; insulin resistance may contribute to blood pressure elevation, but the reverse, whereby sustained haemodynamic load promotes metabolic dysregulation, is equally consistent with the data, and both phenomena may share upstream determinants. Prospective cohort studies with serial ABPM and metabolic measurements are needed to establish temporal relationships.

Second, the covariate set, although broader than in the initial analysis (now including eGFR and HbA1c), does not capture all relevant confounders. Obstructive sleep apnoea is an established determinant of the non-dipping and reverse-dipping phenotypes and is associated with both systemic inflammation and insulin resistance [9]; because formal sleep-study data were unavailable, residual confounding related to OSA cannot be excluded and may be particularly relevant for the circadian-phenotype analyses. Similarly, data on smoking status, physical activity, dietary sodium intake, and medications that influence the TyG index components (statins, metformin, SGLT2 inhibitors, GLP-1 receptor agonists) were not available. The term ‘treatment-naïve’ in this study refers specifically to antihypertensive medications; patients receiving metabolic therapies were not excluded. The assumed causal structure is now made explicit in a conceptual causal diagram (Supplementary Figure S1), but that structure cannot be verified in cross-sectional data; the distinction between confounder and mediator for HbA1c, diabetes, and BMI in the TyG–blood pressure relationship cannot be resolved here, so the adjusted odds ratios are reported as a descriptive conditional association rather than as a total or a direct causal effect. No quantitative bias analysis was undertaken because the assumptions needed to translate an ordinal common odds ratio onto a risk-ratio scale could not be justified for this estimand; unmeasured confounding, reverse causation, selection, and measurement error therefore remain unquantified.

Third, BMI, although available and adjusted for, is a limited measure of adiposity. Waist circumference, waist-to-hip ratio, visceral adiposity index, and imaging-based body composition were not available. Residual confounding attributable to unmeasured adiposity phenotypes cannot be excluded. Although eGFR was incorporated into all multivariable models, albuminuria and other markers of chronic kidney disease staging were unavailable; the full spectrum of renal involvement in the TyG–blood pressure relationship may not, therefore, be adequately captured.

Fourth, ambulatory blood pressure classification was based on a single 24 h recording. The reproducibility of circadian phenotype assignment from a single session is limited; night-to-night variability in the nocturnal decline has been documented, and some individuals may be misclassified [5]. This is a recognised limitation of all single-session ABPM studies and may attenuate the observed associations. The continuous nocturnal systolic dipping percentage was examined as a secondary analysis, but classification of circadian phenotype from a single 24 h session has limited reproducibility and may attenuate the observed associations. Daytime and nighttime periods were defined by fixed clock intervals (06:00–22:00 and 22:00–06:00) rather than by sleep–wake diaries or actigraphy, and information on shift work or atypical sleep schedules was unavailable; misclassification of the circadian phenotype is therefore possible, particularly in individuals whose habitual sleep period differs from the fixed window. Minimum numbers of valid daytime and nighttime readings were not specified separately by the local protocol and could not be recovered retrospectively. In addition, no independent, device-specific validation study for the Echo80 monitor was identified. The AAMI/ESH/ISO Universal Standard specifies how blood-pressure-measuring devices should be validated; compliance with that general protocol does not establish the measurement accuracy of this particular monitor, and the absence of a device-specific validation report is acknowledged as a material limitation of the ambulatory measurements.

Fifth, the reverse- and extreme-dipper subgroups were small (*n* = 63 and *n* = 40, respectively), particularly within the non-elevated BP stratum, and the post hoc pairwise comparisons in these subgroups carry wide uncertainty. No sample-size or precision calculation was carried out in advance; the analysis used all eligible consecutive patients, and the achieved precision is therefore described rather than offered as evidence of adequacy. For the primary association, the 95% confidence interval for the TyG odds ratio ran from 1.34 to 1.83. Precision was considerably lower for the exploratory interaction and for the phenotype-specific estimates: the reverse- and extreme-dipper groups comprise only 63 and 40 participants, respectively, and as few as seven within the non-elevated stratum, so those confidence intervals are wide, and the corresponding estimates are imprecise and should be interpreted with caution.

Sixth, the study was conducted at a single tertiary cardiology centre, and the demographic and metabolic characteristics of the cohort may not be representative of community-based populations. The absence of external replication means that the generalisability of the observed associations to other ethnic, geographic, and clinical settings is unknown. Finally, no longitudinal follow-up data were available; whether or not the observed cross-sectional associations translate into differences in incident cardiovascular events, progression to established hypertension, or target-organ damage could not be evaluated and remains a priority for future research.

## 5. Conclusions

In this ABPM-based cross-sectional study of 723 treatment-naïve individuals, a higher TyG index was associated with ambulatory blood pressure category across the ordered non-elevated-to-hypertension spectrum and, in multinomial analysis, with the reverse- and extreme-dipper circadian phenotypes. The association between the TyG index and blood pressure category differed across circadian phenotypes. These findings are best regarded as characterising cross-sectional associations within a single ABPM cohort rather than as evidence for clinical risk stratification. Confirmation in prospective, multi-centre studies using serial ABPM measurements, formal sleep-apnoea assessment, and longitudinal cardiovascular endpoints is warranted.

## Figures and Tables

**Table 1:** Baseline characteristics according to ambulatory blood pressure category.

Variable	Non-Elevated (*n* = 199)	Elevated (*n* = 243)	Hypertension (*n* = 281)	*p*	*p*-Trend	Hypertension vs. Non-Elevated, Difference (95% CI)
Age, years	46.6 ± 15.3	49.8 ± 16.0	55.3 ± 13.9	<0.001	<0.001	+8.7 (+6.0 to +11.3)
Male sex, *n* (%)	68 (34.2)	96 (39.5)	124 (44.1)	0.089	0.028	+10.0 pp (+1.2 to +18.7)
BMI, kg/m^2^	26.8 ± 2.3	26.5 ± 2.3	26.6 ± 2.2	0.325	0.635	−0.2 (−0.6 to +0.2)
Diabetes mellitus, *n* (%)	48 (24.1)	74 (30.5)	105 (37.4)	0.008	0.002	+13.2 pp (+5.0 to +21.5)
HbA1c, %	5.6 [5.2–6.1]	5.7 [5.3–6.3]	5.7 [5.3–6.3]	0.183	0.145	+0.10 (0.00 to +0.30)
Glucose, mg/dL	91.0 [82.0–103.0]	95.0 [86.0–113.0]	107.0 [90.0–131.0]	<0.001	<0.001	+14 (+10 to +21)
Triglycerides, mg/dL	124.0 [108.0–176.0]	160.0 [118.5–238.5]	162.0 [110.0–253.0]	<0.001	<0.001	+23 (+15 to +69)
HDL-C, mg/dL	47.4 ± 6.9	46.9 ± 7.7	45.7 ± 7.2	0.030	0.015	−1.7 (−3.0 to −0.4)
TyG index	8.8 ± 0.5	9.1 ± 0.5	9.2 ± 0.6	<0.001	<0.001	+0.35 (+0.25 to +0.45)
Creatinine, mg/dL	0.8 ± 0.2	0.8 ± 0.2	0.8 ± 0.2	0.079	0.017	+0.04 (0.00 to +0.08)
eGFR, mL/min/1.73 m^2^	103.0 ± 20.4	99.4 ± 20.3	95.7 ± 18.7	<0.001	<0.001	−7.3 (−10.9 to −3.7)
CRP, mg/L	2.0 [0.8–4.6]	2.4 [1.0–5.0]	2.8 [1.2–5.4]	0.054	0.016	+0.42 (+0.27 to +1.47)
24 h SBP, mmHg	106.6 ± 5.9	121.6 ± 4.1	142.5 ± 9.7	<0.001	<0.001	+35.8 (+34.4 to +37.2)
24 h DBP, mmHg	58.8 ± 3.6	69.7 ± 5.3	83.3 ± 9.7	<0.001	<0.001	+24.4 (+23.2 to +25.7)
Daytime SBP, mmHg	111.8 ± 13.3	125.8 ± 11.2	141.6 ± 12.6	<0.001	<0.001	+29.7 (+27.3 to +32.1)
Daytime DBP, mmHg	64.5 ± 6.9	71.9 ± 7.4	82.8 ± 11.1	<0.001	<0.001	+18.3 (+16.7 to +19.9)
Nighttime SBP, mmHg	100.7 ± 12.0	115.0 ± 13.4	135.3 ± 15.9	<0.001	<0.001	+34.6 (+32.1 to +37.1)
Nighttime DBP, mmHg	56.9 ± 8.9	63.5 ± 9.6	76.5 ± 12.6	<0.001	<0.001	+19.6 (+17.7 to +21.5)
Nocturnal dipping, %	9.8 [4.5–14.3]	8.3 [1.6–14.8]	5.1 [−2.0–10.9]	<0.001	<0.001	−4.9 (−6.5 to −2.7)

Data are mean ± SD, median [IQR], or *n* (%). *p*: overall difference across the three categories (one-way ANOVA, Kruskal–Wallis, or χ^2^ test, as appropriate). *p*-trend: test for a monotonic trend across the ordered categories (Non-Elevated → Elevated → Hypertension)—Jonckheere–Terpstra for continuous variables and Cochran–Armitage for categorical variables. BP categories were defined according to the 2024 European Society of Cardiology (ESC) guidelines. TyG index = ln[triglycerides (mg/dL) × glucose (mg/dL)/2]. eGFR, estimated glomerular filtration rate; SBP/DBP, systolic/diastolic blood pressure. Effect estimates in the final column are the difference between the Hypertension and Non-Elevated categories: mean difference with Welch 95% CI for approximately normally distributed variables; Hodges–Lehmann location-shift estimate with bootstrap 95% CI (4000 resamples) for skewed variables; and absolute risk difference in percentage points (pp) with a normal-approximation (Wald) 95% CI for categorical variables. These are descriptive contrasts and are not adjusted for covariates or for multiplicity.

**Table 2:** Metabolic profile across circadian blood pressure phenotypes within each ambulatory BP category.

Variable	Dipper	Non-Dipper	Reverse	Extreme	*p*
A. Non-Elevated BP (dipper, *n* = 89; non-dipper, *n* = 94; reverse, *n* = 9; extreme, *n* = 7)					
TyG index	8.8 ± 0.4 ^b^	8.8 ± 0.5 ^b^	9.8 ± 0.1 ^a^	9.8 ± 0.3 ^a^	<0.001
HbA1c, %	5.5 [5.1–5.8] ^c^	5.5 [5.3–6.2] ^ac^	6.4 [6.3–7.2] ^b^	7.2 [6.3–7.8] ^ab^	<0.001
Glucose, mg/dL	89.0 [82.0–98.0] ^b^	89.0 [80.2–102.8] ^b^	127.0 [116.0–156.0] ^a^	127.0 [119.5–146.0] ^a^	<0.001
Triglycerides, mg/dL	119.0 [108.0–164.0] ^b^	114.0 [108.0–161.0] ^b^	289.0 [238.0–310.0] ^a^	253.0 [241.5–301.0] ^a^	<0.001
HDL-C, mg/dL	47.1 ± 6.4 ^a^	48.6 ± 6.3 ^a^	45.4 ± 9.9 ^ab^	39.1 ± 10.3 ^b^	0.003
CRP, mg/L	2.0 [0.7–3.7] ^a^	1.6 [0.7–5.1] ^a^	3.9 [3.0–5.2] ^a^	3.7 [2.4–4.5] ^a^	0.123
B. Elevated BP (dipper, *n* = 91; non-dipper, *n* = 120; reverse, *n* = 21; extreme, *n* = 11)					
TyG index	8.9 ± 0.5 ^b^	9.0 ± 0.5 ^b^	9.6 ± 0.4 ^a^	9.6 ± 0.6 ^a^	<0.001
HbA1c, %	5.5 [5.3–6.1] ^b^	5.7 [5.3–6.1] ^b^	6.7 [5.9–7.7] ^a^	7.4 [6.9–7.8] ^a^	<0.001
Glucose, mg/dL	91.0 [84.0–102.0] ^c^	94.0 [86.0–111.0] ^bc^	126.0 [117.0–140.0] ^a^	108.0 [105.5–112.5] ^ab^	<0.001
Triglycerides, mg/dL	149.0 [113.0–192.0] ^b^	157.0 [112.0–224.0] ^b^	257.0 [160.0–289.0] ^a^	308.0 [225.5–345.5] ^a^	<0.001
HDL-C, mg/dL	47.7 ± 6.9 ^a^	47.2 ± 7.3 ^a^	45.0 ± 9.6 ^ab^	39.7 ± 11.0 ^b^	0.007
CRP, mg/L	2.0 [0.7–4.4] ^a^	2.3 [0.8–5.7] ^a^	3.1 [2.0–5.0] ^a^	3.7 [1.8–4.5] ^a^	0.236
C. Hypertension (dipper, *n* = 65; non-dipper, *n* = 161; reverse, n = 33; extreme, *n* = 22)					
TyG index	9.1 ± 0.6 ^b^	9.1 ± 0.5 ^b^	9.6 ± 0.5 ^a^	9.6 ± 0.5 ^a^	<0.001
HbA1c, %	5.7 [5.2–6.0] ^b^	5.5 [5.3–6.1] ^b^	6.1 [5.7–7.3] ^a^	6.8 [6.0–7.6] ^a^	<0.001
Glucose, mg/dL	96.0 [86.0–119.0] ^b^	101.0 [88.0–117.0] ^b^	144.0 [122.0–168.0] ^a^	136.0 [115.0–148.0] ^a^	<0.001
Triglycerides, mg/dL	150.0 [108.0–254.0] ^a^	139.0 [109.0–243.0] ^a^	211.0 [155.0–268.0] ^a^	227.5 [163.8–326.2] ^a^	0.019
HDL-C, mg/dL	46.2 ± 7.2 ^ab^	46.8 ± 6.5 ^a^	43.0 ± 8.0 ^bc^	40.6 ± 8.5 ^c^	<0.001
CRP, mg/L	2.0 [0.7–3.1] ^b^	3.1 [1.2–6.0] ^a^	3.5 [2.1–4.8] ^ab^	3.4 [2.5–4.5] ^ab^	0.014

Continuous data are mean ± SD (TyG index, HDL-C) or median [IQR] (HbA1c, glucose, triglycerides, CRP). The *p*-value is the overall comparison of the four circadian phenotypes within each BP category (one-way ANOVA or Kruskal–Wallis test, as appropriate). Superscript letters denote the post hoc compact letter display: within each BP category and each row, values that do not share a common superscript letter differ significantly, whereas values sharing a letter do not (pairwise comparisons: Tukey’s HSD for TyG index and HDL-C; Dunn’s test with Bonferroni correction for HbA1c, glucose, triglycerides and CRP; α = 0.05). Letter “a” is assigned to the highest value. Reverse- and extreme-dipper subgroups are small within the non-elevated BP stratum (*n* = 9 and *n* = 7, respectively); corresponding post hoc results should be interpreted with caution.

**Table 3:** Ordinal (proportional-odds) logistic regression for the BP-category progression. (Non-Elevated → Elevated → Hypertension).

Variable	OR	95% CI	*p*
Age (per SD)	1.49	1.23–1.79	<0.001
Male sex	1.29	0.97–1.72	0.078
HbA1c (per SD)	0.91	0.79–1.06	0.218
eGFR (per SD)	1.07	0.89–1.29	0.466
BMI (per SD)	0.90	0.78–1.03	0.121
TyG index (per SD)	1.57	1.34–1.83	<0.001

Proportional-odds ordinal logistic regression with the ordered three-level outcome (Non-Elevated, *n* = 199 < Elevated, *n* = 243 < Hypertension, *n* = 281). Each OR shows the odds of being in a higher BP category per 1-SD increment (or, for sex, male vs. female). Adjusted proportional-odds ordinal logistic regression model, with ambulatory BP category (Non-Elevated, Elevated, Hypertension) as the ordered outcome. Continuous predictors are expressed per one-standard-deviation (SD) increment. The proportional-odds assumption was assessed using the Brant test (Supplementary Table S7).

**Table 4:** TyG index × circadian phenotype interaction within the ordinal BP-category model.

Term	OR	95% CI	*p*
Main effects			
TyG index (per SD, in dippers)	1.69	1.28–2.21	<0.001
Non-dipper vs. dipper	1.72	1.26–2.36	0.001
Reverse-dipper vs. dipper	3.36	1.49–7.59	0.004
Extreme-dipper vs. dipper	3.63	1.41–9.33	0.008
Interaction terms			
TyG × Non-dipper	0.96	0.68–1.35	0.811
TyG × Reverse-dipper	0.45	0.24–0.88	0.019
TyG × Extreme-dipper	0.55	0.27–1.12	0.101

Interaction terms were obtained by adding a TyG index × circadian phenotype term to the proportional-odds ordinal model (outcome: ambulatory BP category; covariates: age, sex, HbA1c, eGFR, BMI). Main effects are the per-SD TyG odds ratio within the reference (dipper) phenotype and the odds ratio for each phenotype relative to dippers at the mean TyG value. Each interaction OR is the ratio of the per-SD TyG effect in that phenotype relative to that of dippers (reference); an OR below 1 denotes an attenuated TyG–BP association. Overall interaction: likelihood-ratio χ^2^ = 8.26, df = 3, *p* = 0.041. This analysis is exploratory.

## Data Availability

The complete R analysis script, variable dictionary, file manifest, session log (R version, package versions and random seeds), environment lock file, the exact machine-generated aggregate outputs of the source-data run, and an anonymised synthetic analytical dataset have been deposited in a public repository and are available without restriction at https://doi.org/10.5281/zenodo.21951990. The same files are additionally supplied as Supplementary Materials with this submission. The synthetic dataset permits the analysis code to be executed end to end and the analytical workflow to be audited structurally; it reproduces the direction, the statistical significance and the approximate magnitude of the reported associations, but it does not reproduce the published estimates digit for digit, and no claim of exact numerical reproducibility from synthetic data is made. Exact numerical reproduction requires the source records, which derive from the electronic medical records of a single institution and cannot be shared publicly; requests for access to the de-identified source data will be considered by the institutional data access committee, subject to institutional confidentiality regulations. So that the reported figures can nevertheless be verified line by line, the machine-generated aggregate outputs of the source-data run are deposited alongside the code, and every estimate in the manuscript, tables and Supplementary Materials can be traced to a corresponding output line.

## Supplementary Materials

The following supporting information can be downloaded at: https://www.mdpi.com/article/10.3390/diagnostics16172754/s1, Executable Package. Figure S1. Conceptual causal framework for covariate selection and interpretation of adjusted associations; Table S1. Spearman rank correlations between the TyG index and metabolic and renal variables; Table S2. Post-hoc pairwise comparisons of the TyG index across ambulatory blood pressure categories; Table S3. Sensitivity analysis: proportional-odds ordinal model after excluding patients with diabetes mellitus; Table S4. Distribution of circadian blood pressure phenotypes across ambulatory blood pressure categories; Table S5. Multinomial logistic regression of circadian phenotype on the TyG index (reference: dipper); Table S6. Multicollinearity assessment (variance inflation factors) of the fully adjusted ordinal model; Table S7. Brant test of the proportional-odds (parallel-lines) assumption; Table S8. Assessment of non-linearity in the relationship between the TyG index and the circadian outcome; Table S9. Missing data before complete-case analysis; Table S10. Nested adjustment sequence for the primary ordinal model; Table S11. Model-based adjusted differences in the TyG index between ambulatory blood pressure categories within each circadian phenotype.

## Abbreviations

ABPM	Ambulatory Blood Pressure Monitoring
ANOVA	Analysis of Variance
BMI	Body Mass Index
BP	Blood Pressure
CI	Confidence Interval
CKD-EPI	Chronic Kidney Disease Epidemiology Collaboration
CRP	C-Reactive Protein
DBP	Diastolic Blood Pressure
eGFR	estimated Glomerular Filtration Rate
ESC	European Society of Cardiology
GLP-1	Glucagon-Like Peptide-1
HbA1c	Glycated Haemoglobin
HDL-C	High-Density Lipoprotein Cholesterol
IQR	Interquartile Range
OR	Odds Ratio
OSA	Obstructive Sleep Apnoea
SBP	Systolic Blood Pressure
SD	Standard Deviation
SGLT2	Sodium–Glucose Cotransporter-2
STROBE	Strengthening the Reporting of Observational Studies in Epidemiology
TyG	Triglyceride–Glucose (index)
VIF	Variance Inflation Factor
