# Supplementary material for: Association of the Triglyceride–Glucose Index with Ambulatory Blood Pressure Categories and Circadian Blood Pressure Phenotypes in Untreated Adults: A Single-Centre, ABPM-Based Retrospective Cross-Sectional Study
Source: Diagnostics (Basel). 2026 Aug 27;16(17):2754. doi: 10.3390/diagnostics16172754 (PMC13565201; doi:10.3390/diagnostics16172754)
Supplement: Supplementary file 1 [file diagnostics-16-02754-s001.zip › Supplementary Tables.pdf]

Table S1. Spearman rank correlations between the TyG index and metabolic and renal variables

Variable	Spearman ρ	p
Triglycerides	+0.870	<0.001
Fasting glucose	+0.686	<0.001
HbA1c	+0.265	<0.001
HDL-C	-0.288	<0.001
eGFR	-0.253	<0.001
C-reactive protein	+0.104	0.005
Body mass index	+0.014	0.699

Spearman rank correlation coefficients (n = 723). BMI, body mass index; eGFR, estimated glomerular filtration rate; HDL-C, high-density lipoprotein cholesterol.

Table S2. Post-hoc pairwise comparisons of the TyG index across ambulatory blood pressure categories

Comparison	Adjusted p
Non-elevated vs Elevated	<0.001
Non-elevated vs Hypertension	<0.001
Elevated vs Hypertension	0.015

Tukey HSD test following one-way ANOVA (the TyG index was approximately normally distributed and is summarised as mean ± SD in Table 1). All three pairwise contrasts across the ordered BP categories were statistically significant.

Table S3. Sensitivity analysis: proportional-odds ordinal model after excluding patients with diabetes mellitus

Variable	OR	95% CI	p
Age (per SD)	1.47	1.16–1.85	0.001
Male sex	1.59	1.13–2.25	0.008
HbA1c (per SD)	1.01	0.85–1.19	0.914
eGFR (per SD)	1.08	0.86–1.36	0.511
BMI (per SD)	0.96	0.81–1.14	0.648
**TyG index (per SD)**	**1.45**	**1.22–1.73**	**<0.001**

Proportional-odds ordinal logistic regression in participants without diabetes mellitus (n = 496). Outcome: ambulatory BP category (Non-elevated < Elevated < Hypertension). The TyG index gradient across categories persisted (8.7, 8.9, and 9.0). Continuous predictors are expressed per 1-standard-deviation increment. OR, odds ratio; CI, confidence interval.

Table S4. Distribution of circadian blood pressure phenotypes across ambulatory blood pressure categories

Circadian phenotype	Non-elevated	Elevated	Hypertension	Total
Dipper	89	91	65	245
Non-dipper	94	120	161	375
Reverse-dipper	9	21	33	63
Extreme-dipper	7	11	22	40
**Total**	**199**	**243**	**281**	**723**

Values are counts of participants (n = 723). χ^2 test across the distribution, $p < 0.001$.

Table S5. Multinomial logistic regression of circadian phenotype on the TyG index (reference: dipper)

A. Non-dipper vs Dipper

Variable	OR	95% CI	p
TyG index (per SD)	1.02	0.84–1.24	0.856
Age (per SD)	1.15	0.92–1.45	0.228
Male sex	0.88	0.62–1.23	0.449
HbA1c (per SD)	1.12	0.93–1.36	0.230
eGFR (per SD)	1.04	0.82–1.31	0.773
BMI (per SD)	1.07	0.90–1.26	0.458
Elevated BP	1.23	0.82–1.84	0.328
Hypertension	2.22	1.44–3.41	<0.001

B. Reverse-dipper vs Dipper

Variable	OR	95% CI	p
TyG index (per SD)	2.90	2.06–4.08	<0.001
Age (per SD)	0.85	0.57–1.28	0.447
Male sex	1.47	0.78–2.75	0.231
HbA1c (per SD)	1.49	1.11–2.00	0.008
eGFR (per SD)	0.61	0.42–0.88	0.009
BMI (per SD)	0.96	0.71–1.28	0.760
Elevated BP	1.68	0.68–4.16	0.260
Hypertension	2.86	1.17–6.99	0.022

C. Extreme-dipper vs Dipper

Variable	OR	95% CI	p
TyG index (per SD)	2.77	1.87–4.10	<0.001
Age (per SD)	0.55	0.34–0.89	0.015
Male sex	1.09	0.51–2.32	0.828
HbA1c (per SD)	1.96	1.45–2.65	<0.001
eGFR (per SD)	0.54	0.35–0.83	0.005
BMI (per SD)	1.26	0.91–1.74	0.169
Elevated BP	1.21	0.41–3.59	0.725
Hypertension	3.36	1.21–9.35	0.020

Multinomial logistic regression (n = 723) with the four circadian phenotypes as the outcome and the dipper phenotype as the reference. Each model was adjusted for age, sex, HbA1c, eGFR, BMI, and ambulatory BP category. Continuous predictors are expressed per 1-standard-deviation increment. OR, odds ratio; CI, confidence interval.

Table S6. Multicollinearity assessment (variance inflation factors) of the fully adjusted ordinal model

Variable	VIF
Age	1.81
Male sex	1.03
HbA1c	1.09
eGFR	1.83
BMI	1.01
TyG index	1.16

Variance inflation factors from the fully adjusted model. All values were below 2, indicating no problematic multicollinearity.

Table S7. Brant test of the proportional-odds (parallel-lines) assumption

A. Primary ordinal model

Term	χ^2	p
Age	1.48	0.223
Male sex	0.01	0.922
HbA1c	0.19	0.667
eGFR	0.21	0.647
BMI	1.82	0.177
TyG index	2.72	0.099
**Omnibus**	**6.41**	**0.379**

Brant test of the proportional-odds assumption for the primary model (omnibus $\chi^2 = 6.41$, df = 6, p = 0.379). No term reached statistical significance, providing no evidence against the proportional-odds assumption. Per-term tests carry 1 degree of freedom each.

B. Expanded model including circadian phenotype and TyG × phenotype interaction terms

Term	χ^2	p
Age	1.74	0.187
Male sex	0.01	0.935
HbA1c	0.58	0.447
eGFR	0.17	0.681
BMI	1.46	0.227
TyG index	0.32	0.571
Non-dipper vs dipper	0.61	0.435
Reverse-dipper vs dipper	1.05	0.305
Extreme-dipper vs dipper	0.02	0.895
TyG × non-dipper	1.04	0.308
TyG × reverse-dipper	1.71	0.191
TyG × extreme-dipper	0.63	0.428
**Omnibus**	**13.44**	**0.338**

Brant test applied to the expanded model reported in Table 4 (omnibus $\chi^2 = 13.44$, df = 12, p = 0.338). No individual term reached statistical significance (all $p \geq 0.187$), providing no evidence against the proportional-odds assumption for the expanded model either. Per-term tests carry 1 degree of freedom each.

Table S8. Assessment of non-linearity in the relationship between the TyG index and the circadian outcome

A. Nocturnal systolic dipping percentage as the outcome, TyG index as the exposure

Term for the TyG index	β	95% CI	p
Linear (per SD)	−0.67	−1.45 to 0.12	0.096
Quadratic term (per SD²)	−0.16	−0.80 to 0.48	0.623
Restricted cubic spline, non-linear term (per SD)	−2.92	−5.99 to 0.16	0.063

Multivariable linear regression (n = 723); outcome: nocturnal systolic dipping percentage; exposure: TyG index, entered (i) linearly per 1-standard-deviation increment, (ii) as a linear plus quadratic term, and (iii) as a restricted cubic spline with three knots placed at the 10th, 50th and 90th percentiles of the TyG distribution (8.42, 8.95 and 9.84 on the raw scale); the spline and quadratic terms were fitted on the standardised TyG index, so their coefficients are per 1-SD unit; all models adjusted for age, sex, HbA1c, eGFR, BMI and ambulatory BP category. This is the direction of the primary scientific question. Neither the quadratic term nor the spline non-linear term reached statistical significance (F = 3.46 on 1 and 713 degrees of freedom for the spline term), and the model R^2 rose only from 0.064 to 0.069. There is therefore no statistically significant evidence that the expected nocturnal dipping percentage varies non-linearly with the TyG index.

B. Adjusted mean nocturnal dipping percentage across the TyG spectrum

TyG index	Adjusted mean nocturnal systolic dipping, %
8.0	6.1
8.5	7.3
9.0	7.8
9.5	6.9
10.0	5.1
10.5	3.3

Predictions from the restricted cubic spline model in panel A, obtained by g-computation: the TyG index was set to each listed value for every participant in turn and the model predictions were averaged over the observed distribution of the remaining covariates. The predicted values vary only modestly across the observed TyG range, and the confidence limits around the non-linear term in panel A include no departure from linearity; these predictions are therefore descriptive and no particular functional shape is claimed.

C. Circadian phenotype as the outcome: flexible versus linear TyG term

Model for circadian phenotype	Log-likelihood	Likelihood-ratio χ^2 (df)	p
Linear TyG term	−689.91	—	—
Restricted cubic spline TyG term	−689.52	0.79 (3)	0.853

Multinomial logistic regression (n = 723) with the four circadian phenotypes as the outcome (reference: dipper), adjusted for age, sex, HbA1c, eGFR, BMI and ambulatory BP category. The model containing a restricted cubic spline in the TyG index (three knots, as in panel A) was compared with the corresponding model containing a linear TyG term by a likelihood-ratio test. Adding the flexible term did not improve fit, so a linear TyG term is adequate for the categorical phenotype outcome.

D. Reverse-direction descriptive model: TyG index as the outcome, nocturnal dipping as the exposure

Term for nocturnal dipping	β	95% CI	p
Linear (per SD)	−0.025	−0.062 to 0.013	0.198
Quadratic term	0.062	0.039 to 0.085	<0.001
Restricted cubic spline, non-linear term	0.226	0.145 to 0.308	<0.001

Nocturnal systolic dipping, %	Adjusted mean TyG index
−10	9.25
−5	9.14
0	9.04
+5	8.98
+10	8.97
+15	9.03
+20	9.10
+25	9.18

Multivariable linear regression (n = 723); outcome: TyG index; exposure: nocturnal systolic dipping percentage entered as a linear plus quadratic term and, separately, as a restricted cubic spline with three knots at the 10th, 50th and 90th percentiles of the dipping distribution (−6.0%, 7.5% and 18.7%); adjusted for age, sex, HbA1c, eGFR, BMI and ambulatory BP category. Adding the non-linear term increased the model R^2 from 0.184 to 0.217. The second table gives predictions from that model by g-computation. Because the direction of the conditional regression is reversed relative to panels A to C, this panel is descriptive only: evidence that the mean TyG index varies non-linearly across dipping values does not demonstrate that the expected dipping percentage varies non-linearly across TyG values. It is not used to confirm the primary estimand and it is not the justification for modelling circadian phenotype categorically.

Table S9. Missing data before complete-case analysis

Variable	Missing, n	Missing, %
HbA1c	15	1.7
Triglycerides	11	1.2
Fasting glucose	9	1.0
Body mass index	5	0.6
eGFR	3	0.3
**Participants with any missing value**	**43**	**4.9**

Missingness was assessed among the 885 potentially eligible antihypertensive-naïve adults who remained after exclusion of participants with prior hypertension or antihypertensive treatment (Figure 1). Denominator for each percentage is 885. Variables not listed (age, sex, ambulatory blood pressure values, creatinine, HDL-cholesterol and C-reactive protein) were complete. No single variable was missing in more than 2% of records, and the variable-specific counts sum exactly to the 43 participants excluded for missing data, so no participant had more than one missing value. Given this low and non-overlapping missingness, a complete-case analysis was performed and no imputation was undertaken. Because the excluded records were themselves incomplete for the variables required, a formal comparison of included with excluded participants was not undertaken. eGFR, estimated glomerular filtration rate.

Table S10. Nested adjustment sequence for the primary ordinal model

Model	TyG index, OR per SD	95% CI	p
M0 TyG index alone (unadjusted)	1.64	1.42–1.89	<0.001
M1 + age, sex	1.51	1.30–1.75	<0.001
M2 + eGFR, BMI	1.53	1.32–1.78	<0.001
M3 + HbA1c (primary model)	1.57	1.34–1.83	<0.001
M3b + diabetes instead of HbA1c	1.57	1.34–1.86	<0.001

Proportional-odds ordinal logistic regression (n = 723); outcome: ambulatory BP category (Non-elevated < Elevated < Hypertension). Models are presented as a transparent nested sequence rather than as an assertion that any particular variable is a confounder. M1 adds the variables that can reasonably be regarded as antecedent to the exposure; M2 adds renal function and adiposity; M3 adds HbA1c, whose status as confounder, intermediate or marker of the same metabolic state cannot be resolved in cross-sectional data; M3b substitutes the binary diabetes indicator for HbA1c. The estimate for the TyG index changes by less than 5% across the sequence, so the reported association does not depend on whether the potentially intermediate metabolic variables are conditioned on. OR, odds ratio; CI, confidence interval.

Table S11. Model-based adjusted differences in the TyG index between ambulatory blood pressure categories within each circadian phenotype

Circadian phenotype	Contrast	Adjusted mean difference in TyG index (95% CI)	p
Dipper	Elevated vs Non-elevated	+0.18 (+0.04 to +0.32)	0.014
Dipper	Hypertension vs Non-elevated	+0.31 (+0.15 to +0.47)	<0.001
Dipper	Hypertension vs Elevated	+0.13 (−0.03 to +0.29)	0.100
Non-dipper	Elevated vs Non-elevated	+0.22 (+0.09 to +0.36)	0.001

Circadian phenotype	Contrast	Adjusted mean difference in TyG index (95% CI)	p
Non-dipper	Hypertension vs Non-elevated	+0.28 (+0.16 to +0.40)	<0.001
Non-dipper	Hypertension vs Elevated	+0.06 (−0.06 to +0.17)	0.339
Reverse-dipper	Elevated vs Non-elevated	−0.26 (−0.64 to +0.12)	0.186
Reverse-dipper	Hypertension vs Non-elevated	−0.24 (−0.60 to +0.12)	0.189
Reverse-dipper	Hypertension vs Elevated	+0.02 (−0.25 to +0.28)	0.901
Extreme-dipper	Elevated vs Non-elevated	−0.14 (−0.60 to +0.32)	0.552
Extreme-dipper	Hypertension vs Non-elevated	−0.28 (−0.69 to +0.14)	0.190
Extreme-dipper	Hypertension vs Elevated	−0.14 (−0.49 to +0.22)	0.447

Linear regression ($n = 723$) with the TyG index as the outcome and an ambulatory BP category × circadian phenotype interaction, adjusted for age, sex, eGFR and BMI (HbA1c is not included because it is a glycaemic co-marker of the outcome variable). Each row is a model-based contrast of adjusted mean TyG values, and corresponds to one of the 12 unadjusted pairwise comparisons displayed in Figure 2; the two sets of results agree in direction throughout. The contrasts are not corrected for multiplicity and remain exploratory, but reporting them with confidence intervals makes the reverse- and extreme-dipper panels interpretable: the intervals there are wide and include differences of both signs, so the absence of statistical significance reflects limited precision rather than demonstrated homogeneity. Overall interaction $F = 2.47$ on 6 and 707 degrees of freedom, $p = 0.023$; model $R^2 = 0.260$. CI, confidence interval.
