# Supplementary material for: Association of the Triglyceride–Glucose Index with Ambulatory Blood Pressure Categories and Circadian Blood Pressure Phenotypes in Untreated Adults: A Single-Centre, ABPM-Based Retrospective Cross-Sectional Study
Source: Diagnostics (Basel). 2026 Aug 27;16(17):2754. doi: 10.3390/diagnostics16172754 (PMC13565201; doi:10.3390/diagnostics16172754)
Supplement: Supplementary file 1 [file diagnostics-16-02754-s001.zip › Supplementary Figure S1.pdf]

Figure S1. Conceptual causal framework for covariate selection and interpretation of adjusted associations

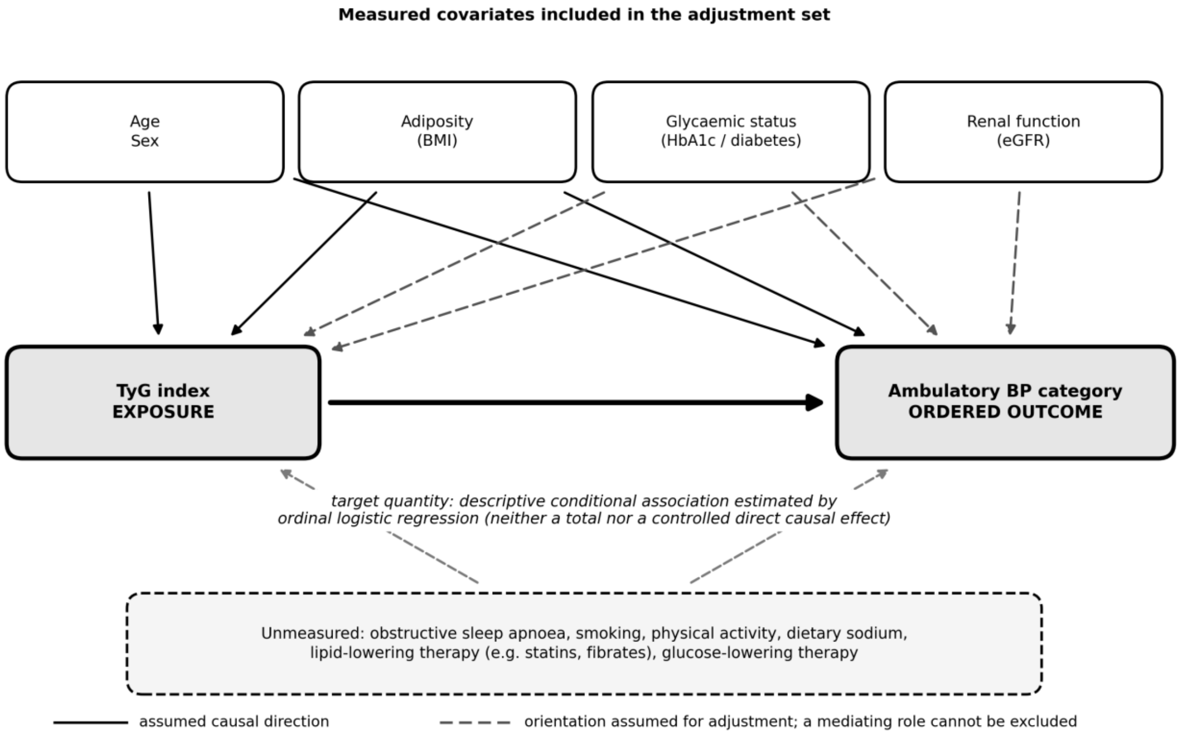

Solid arrows denote directions assumed to be causal. Age and sex are treated as common causes of both the TyG index and ambulatory blood pressure category and are therefore conditioned on as confounders. Glycaemic status (HbA1c / diabetes) and renal function are also conditioned on, but they are not unambiguously antecedent to the exposure: each may act as a confounder, as an intermediate, or as a marker of the same underlying metabolic state. Their edges are drawn dashed and in the orientation assumed when they are used for adjustment; that orientation is an assumption, and a mediating role cannot be excluded on cross-sectional data. Adiposity is treated as a common cause, although a mediating role cannot be excluded. Variables shown in the dashed box were not recorded and represent potential residual confounding. Because the structure shown cannot be verified with the available data, the estimate obtained from the adjustment set is reported as a descriptive conditional association — neither a total causal effect nor a controlled direct effect — and no quantitative bias analysis is attached to it.
